# Science behind children’s handwashing: action study of 9- to 10-years-old elementary school students in Japan

**DOI:** 10.3389/fpubh.2024.1425646

**Published:** 2024-07-18

**Authors:** Asae Oura, Yukiko Naito, Hiroko Yako-Suketomo, Kei Nakata, Masayuki Koyama, Hirofumi Ohnishi

**Affiliations:** ^1^Department of Public Health, School of Medicine, Sapporo Medical University, Sapporo, Hokkaido, Japan; ^2^Public Health Laboratory, School of Allied Health Science, Kitasato University, Sagamihara, Kanagawa, Japan; ^3^Japan Women’s College of Physical Education, Tokyo, Japan

**Keywords:** adenosine triphosphate (ATP)-test, hand washing, health education, elementary school, health literacy

## Abstract

**Background:**

Hand washing instructions for children have been implemented in school education to establish good lifestyle habits. However, repeated hand washing through education from early childhood was common for both teachers and children. If this continues, children might assume they already know how to wash their hands, stop taking handwashing instructions seriously, and become increasingly lax about washing their hands.

**Purpose:**

This study aimed to develop a new handwashing education method for children.

**Methods:**

We applied the adenosine triphosphate (ATP) test to health education on hand washing in elementary schools. This study was conducted as part of a class for elementary school students in October 2023, in Hokkaido, Japan. The subjects were 157 third-grade (9–10 years old) elementary school students. After excluding absent pupils, 147 were included in the analysis.

**Results:**

Both pre- and post-education, ATP values after handwashing were lower than those before handwashing. Following the education, children’s handwashing behavior improved, with an increase in the number of point washed and appropriate timing of handwashing.

**Conclusion:**

The new handwashing education program utilizing the ATP-test succeeded in promoting handwashing behavior among many children. Visualizing handwashing using ATP values was effective in motivating children.

## 1 Introduction

Hand washing is recommended by the WHO as a fundamental measure for infectious diseases in the general public (1). The COVID-19 pandemic has heightened global awareness of hand hygiene through various campaigns. Additionally, climate change, including global warming, is a critical global issue that may contribute to the emergence of new infectious diseases like COVID-19 (2). Therefore, acquiring the habit of hand washing is essential for lifelong prevention of unknown infectious diseases.

Hand washing instructions for children have been implemented with the aim of establishing good lifestyle habits in school education (3, 4). According to curriculum guidelines at elementary schools in Japan, for example, “body cleanliness” and “hand washing” are mentioned in health subjects in the 3rd grade (age range: 9–10 years old) in elementary school (3). In most cases, a homeroom teacher or school nurse teacher using materials available at school such as other commercially available teaching materials (5). The topic is repeatedly covered in early childhood education (5), therefore there should be no surprise for children.

Many previous studies on health education reported self-evaluation, for example, on the topic of hand washing, “self-evaluation based on behavior,” “visual experiment of stain removal using black light” (5). Many reports have only used subjective items, and because of this, few previous articles have used scientific indicators (6–8). The introduction of objective indicators was a challenge for the scientific proof of health education in schools. In 2023, “Active Learning” was emphasized in elementary school education. Japan’s current education policy is aligned with the SDGs that the world is aiming for (9). Children’s questions offer opportunities to explore many of today’s health issues. For example, they may ask, “Do I have to use soap when I wash my hands? Cannot I just use running water?” or “I want clean water to come out when I turn on the tap anywhere in the world!” However, active learning is not being fully utilized. Additionally, since active learning is an educational method focused on student engagement, it tends to be subjective, making objective evaluation crucial for maintaining balance. Even if outcomes are similar, standards and evaluations may vary depending on the child. As a result, there is a new trend in health education that employs objective indicators (8, 10).

Since 2019, our research group has been delivering education on infectious disease prevention to children (11). Owing to the influence of COVID-19, on-site classes were canceled. During the pandemic, we conducted public health activities to encourage children to take the initiative to wash their hands (12). All activities were conducted in collaboration with school education. After the COVID-19 restrictions were lifted, hand washing classes were held at an elementary school. During these classes, we incorporated indicators to quantitatively assess the level of hygiene on children’s hands.

This study introduced objective criteria to determine whether children are good at hand washing their hands. The purpose of this study was to increase children’s interest in hand washing. This study was an action research endeavor (13). Here, the research group collaborated closely with elementary school teachers to implement an action research program. The aim was promptly translate research findings into educational outcomes for children, particularly within the context of school education.

## 2 Materials and methods

This study took place in October 2023 as part of a class for elementary school students in Hokkaido, Japan. The 157 third-grade students (aged 9–10 years). After excluding absent students, the analysis included 147 students.

This study was conducted based on an after-school pilot study (14). Why did the present study incorporate objective indicators into its hand washing program? The first reason was to attract children’s interest. Therefore, we wanted to introduce a real device that is actually used in hygiene management and food sanitation which is not normally available at school. The second reason was to measure hand washing skills using certain passing criteria. Leaving the passing criteria to the children’s judgment and making decisions based only on subjective evaluations. Therefore, adenosine triphosphate (ATP)-testing was introduced to our hand washing program.

ATP is contained in organic matter, including dirt. In this study, therefore, hand cleanliness was assessed by the ATP-testing method conducted with a Lumitester Smart device (Kikkoman) (15). In the method, the amount of ATP is calculated by measuring the luminescence (relative luminescence unit, RLU) emitted when ATP and its decomposition products (ADP and AMP) react with luciferase. This ATP-testing method is widely used in food hygiene management. The Lumitester’s (15) manufacturer has set the ATP standard score at 4000 RLU with a stricter standard of 2000 RLU. Therefore, we also referred to these values in this study. In this study, specialized reagents (Lucipak) were used. The children’s palms, fingers, fingertips, and space between the fingers were wiped after moistening the target areas with running water.

The present study implemented “hand washing” educational program in school classes. The classes lasted for 2 h, and the flow of the education program is shown in Table 1. The ATP levels were measured four times. On the first day, we measured the ATP score before and after hand washing by washing their hands as usual. Following handwashing, adhering to recommendations from the WHO (16) and the Ministry of Health, Labor and Welfare (17), the children were instructed on the proper handwashing technique. The included thorough cleansing of six key areas: the back of the hand, the palm, between the fingers, the thumb, the fingertips, and the wrist. Additionally, they were advised to rinse any remaining soapy foam from their hand under running water for approximately 20 s and to rinse the faucet after washing their hands. We told them to practice at home, because we were going to take the same test the next day. On the second day of re-education, ATP levels were measured again before and after hand washing. After washing their hands on both days, students were asked not to touch anything until the ATP test was completed. There were seven inspectors, including two elementary school-teachers. After all ATP tests, we gave a class on “normal bacteria flora,” to prevent children from “over-washing one’s hands.” Is it feasible to reduce ATP levels to zero? Even if achievable, would such a state sustainable? There were the questions posed to the children. They were instructed to ponder these queries while washing their hands with foaming hand soap provided by our research group, followed by drying with paper towels. Subsequently, they shook the test tube containing the sample 50 times. If an immediate measurement was not obtained, the test was repeated.

**Table 1:** Teaching Plan.

schedule	flow	
1st class	Are your hands clean?	introduction
	↓	
	There is a way to make it visualize	
	↓	
	Let’s check how to wash your hands usually	ATP test (handwashing)
	↓	
	Correct hand washing	education
	There are points to accomplish hand washing!	
		
2nd class	Have you practiced washing your hands? Check it again	ATP test(handwashing)
	↓	
	Is it better to reduce ATP value to zero?	
	↓	
	If you wash hands too much, the important bacteria will also destroyed. Think about when to wash!	education

A questionnaire survey on school education was administered at the end of the class on both days. The questionnaire is presented in Table 2. In this study, individuals were classified as having washed their hands only if they responded affirmatively to the question regarding the timing of handwashing. Those demonstrating correct knowledge about infection prevention were defined as individuals who answered both “Handwashing prevents you from coronavirus” and “Handwashing does not kill all germs.”

**Table 2:** Questionnaire for children.

question			choice
When did you wash your hands yesterday?			
	after reaching school (home)		wash/don't wash/sometime
	after using the restroom		wash/don't wash/sometime
	before eating		wash/don't wash/sometime
	after eating		wash/don't wash/sometime
	before classroom cleaning		wash/don't wash/sometime
	after classroom cleaning		wash/don't wash/sometime
	after touching common things		wash/don't wash/sometime
	after coughing or sneezing		wash/don't wash/sometime
	after playing outside		wash/don't wash/sometime
	other		wash/don't wash
Which parts did you wash?			select all that apply below
	back of hand		
	palm		
	thumb		
	between fingers		
	fingertip		
	wrist		
Do you have a handkerchief today?			yes/no
Do you think the following is correct?			select all that apply below
	Handwashing protects you from coronavirus.		
	Handwashing does not kill all germs.		
	Wash with running water if soap is not available.		
	Wipe your wet hands with clothes if you do not have a handkerchief.		
	It is better to wash your hands frequently since they get dirty.		
Do you want to tell your parents about today at home?			yes/no

The degree of contamination before handwashing varies among children. For instance, if only ATP value were analyzed, the measurement could range from 100,000 to 10,000 among different children. Thus, an index indicating the extent of contamination reduction was necessary. We defined this index as the “percentage change in ATP,” which is calculated as follows:


$$Percentage\;change\;in\;ATP=\left(1-\frac{after\;ATP}{before\;ATP}\right)*100\;\left(\%\right)$$

Statistical analyses were performed using the Statistical Package for Social Science (SPSS version 25, in Japanese). The Wilcoxon signed-rank test and McNemar test were employed to compare pre- and post-education or before- and after-handwashing measures of paired individuals. The significance level was set at less than 0.05. This study was approved by the ethics board of Sapporo Medical University (1-2-22).

## 3 Results

Table 3 shows a comparison of the ATP value pre- and post-education.

Both pre- and post-education, ATP values after handwashing were significantly lower than before handwashing (*p* < 0.001). Moreover, post-education, the ATP value after handwashing remained lower than pre-education (*p* < 0.001). Additionally, before handwashing, the ATP value post-education was significantly lower than pre-education (*p* < 0.001).

Pre-education, pupils with 4,000 RLU or less before handwashing were 2 (1.3%), after handwashing were 105 (70.5%) (*p* < 0.001). Post-education, students with 4,000 RLU or less before handwashing were 20 (13.4%), and after handwashing were 132 (88.6%) (*p* < 0.001). Both before and after handwashing, the number of pupils with 4,000 RLU or less post-education was significantly higher than pre-education (*p* < 0.001).

Pre-education, pupils with 2000 RLU or less before handwashing were 0 (0%), and after hand washing, were 58 (38.9%). Post-education, pupils with 2000 RLU or less before hand washing were 6 (4.0%), and after hand washing were 100 (67.1%) (*p* < 0.001). After handwashing, the number of pupils with 2000 RLU or less post-education was significantly higher than pre-education (*p* < 0.001).

Table 4 shows comparison of children’s hand washing behaviors between pre- and post-education. Post-education, the frequency of handwashing before eating (*p* = 0.01), after eating (*p* = 0.01), before cleaning in a classroom (*p* = 0.01), after cleaning in a classroom (*p* = 0.02), after touching common surfaces (*p* < 0.01), and after coughing or sneezing (*p* < 0.01) was higher than pre-education. Furthermore, the frequency of washing specific areas such as the back of the hand (*p* < 0.01), the thumb (*p* < 0.01), between fingers (*p* < 0.01), fingertips (*p* < 0.01), wrists (*p* < 0.01), and six spots (*p* < 0.01) was higher post-education compared to pre-education. Additionally, the belief that “Handwashing prevents you from coronavirus” (*p* = 0.02), and the understanding that “It is better to wash your hands frequently to prevent contamination” (*p* < 0.01) were more prevalent post-education compared to pre-education.

**Table 3:** Comparison between pre- and post-education: ATP-value.

		Pre-education		Post-education		*p*-value*1	*p*-value*2	*p*-value*3	*p*-value*4
		Before hand washing	After hand washing	Before hand washing	After hand washing				
		*n* = 147	*n* = 147	*n* = 147	*n* = 147				
ATP	measured value	27691.0	2554.0	11886.0	1279.5	<0.001	<0.001	<0.001	<0.001
		[15222, 42988]	[1377.5, 5059.5]	[5872, 23632]	[722.3, 2473.8]				
	<4000	2 (1.3%)	105 (70.5%)	20 (13.4%)	132 (88.6%)	<0.001	<0.001	<0.001	<0.001
	<2000	0 (%)	58 (38.9%)	6 (4.0%)	100 (67.1%)	-	<0.001	-	<0.001

median[25%quartile, 75%quartile] or number(%).

p-value*1: before hand washing v.s. after hand washing in pre-education.

p-value*2: before hand washing v.s. after hand washing in post-education.

p-value*3: comparison between pre and post education, before hand washing.

p-value*4: comparison between pre and post education, after hand washing.

**Table 4:** Comparison between pre and post education:children's behavior.

		Pre-education	Post-education	*p*
		First day	Secound day	
		*n* =147	*n* =147	
Gender	male	62 (41.6%)		
Percentage change in ATP (%)		85.2±15.2	83.2±16.1	0.10
When did you wash your hands yesterday?				
	after reaching school (home)	116 (81.1%)	125(87.4%)	0.09
	after using the restroom	129 (87.2%)	129 (87.2%)	1.00
	before eating	122 (84.1%)	133 (91.7%)	0.01
	after eating	48 (33.3%)	64 (44.4%)	0.01
	before classroom cleaning	20 (14.2%)	33(23.4%)	0.01
	after classroom cleaning	56 (40.9%)	70(51.1%)	0.02
	after touching some common things	62 (42.5%)	88 (60.3%)	<0.01
	after coughing or sneezing	50 (35.0%)	71 (49.7%)	<0.01
	after playing outside	137 (94.5%)	139 (95.9%)	0.69
	other	116 (82.3%)	117 (83.0%)	1.00
Which parts did you wash?				
	back of hand	120 (84.5%)	135 (95.1%)	<0.01
	palm	133(93.7%)	139 (97.9%)	0.07
	thumb	112(78.9%)	134 (94.4%)	<0.01
	between fingers	117 (82.4%)	135 (95.1%)	<0.01
	fingertip	96(67.6%)	133 (93.7%)	<0.01
	wrist	106 (74.6%)	133 (93.7%)	<0.01
	wash hands in 6 spots	64 (46.0%)	124 (89.2%)	<0.01
Do you have a handkerchief today?				
	yes	96 (78.0%)	130 (83.7%)	0.07
Do you think the following is correct?				
1	Handwashing protects you from coronavirus.	91(64.5%)	104 (73.8%)	0.02
2	Handwashing does not kill all germs.	76(53.9%)	86 (59.6%)	0.20
3	Wash with running water if soap is not available.	15(10.6%)	12 (8.5%)	0.55
4	Wipe your wet hands with clothes if you do not have a handkerchief.	8 (5.7%)	11 (7.8%)	0.51
5	It is better to wash your hads frequently since they get dirty.	117 (83.0%)	96(68.1%)	<0.01
	correct knowledge about infections diseases*	52 (36.9%)	64 (45.4%)	0.05
Do you want to tell your parents about today at home?				
	yes	114 (90.5%)	116 (92.1%)	0.73

mean±SD or Number of person (%).

## 4 Discussion

Most previous studies on handwashing have been conducted among health professionals and students, primarily focusing on knowledge (19, 20). Although there have been reports (21) on food hygiene among university students, to our knowledge, hand hygiene education utilizing objective indicators such as the ATP test has not been implemented in elementary schools.

The class processed smoothly. On the second day, the children understood what was going to happen and were seated on time, waiting for the survey to begin. We observed that the children were interested and wanted to participate actively. Elementary teachers also participated in the survey and took notes in their capacity as researchers. Communication between researchers and participants, which is an important core of action studies, led to the success of the investigation (13, 22, 23).

The educational effect of this study was high. For example, a low the ATP value may be low before hand washing on the second day because many children had already washed their hands prior to the ATP test. Post-education, since the children’s hands were clean even before class started, the ability to remove contamination was diminished. However, this is a food hygiene standard, not a standard for the general public. If pupils wash their hands for a long time, they may become germophobia. In order to reduce that risk, we included talking about “normal bacteria flora” in the present education program. Consequently, the effects of the education were observed. In general prevention awareness, they only tell people “to wash one’s hands” (16, 17). However, a few products mentioned washing excessively. Active learning is currently one of the main focuses of education in Japan (3, 4). Therefore, it is important for children to realize that excessive washing is not good. In future studies, we plan to emphasize the that hands can become contaminated immediately after washing.

The effects of education on children’s behaviors were also observed. Post-education, many children wash other areas of their hands beside their palms. A previous report (24) has shown that children were not washing their hands sufficiently. The results of the pre-education test were similar to those of a previous study (24). A similar positive effect of education was observed when the children washed their hands. Pre-education, many pupils washed their hands when they returned from the outside, but many pupils did not wash their hands when they were contaminated, such as after cleaning. After the education program, more children started to wash their hands after cleaning as well. Educational effects were also observed in terms of knowledge. In the present study, understanding of hand washing prevention increased post-education. This finding indicates that children can easily absorb knowledge. We intend to continue the survey next year, focusing on the children surveyed in this study as well as those who will be 9–10 years old at that time. Our aim is to monitor any changes in the children’s behavior over time. We plan to investigate whether the children who participated in this program continue to maintain regular handwashing habits.

If the handwashing awareness program we developed proves to be effective in sustaining children’s interest and promoting regular handwashing, it could serve as a valuable tool not only for infection prevention but also for broader hygiene education, potentially contributing to global hygiene initially (1, 9). Aligned with the goals of the SDGs and Japanese education, our overarching aim is to foster children’s curiosity and encourage them to take independent action. This study successfully achieved that goal through collaborative efforts between researchers and elementary school teachers.

Some limitations of this study should be noted. First, no control group was included. Therefore, the effects of education could not be accurately measured. Second, the sample size was small. Therefore, it is necessary to increase the sample size in future studies. Third, the study was conducted at an elementary school in Sapporo, which is why it was not representative. Fourth, the concept of “overwashing” has not been explicitly defined in this study. During the pre-education period, some children washed their hands repeatedly with hand soap for approximately 5–10 min, while others did so merely because they had touched a pencil. The phenomenon of children washing their hands excessively requires further exploration and clarification.

In conclusion, a handwashing education program using the ATP test succeeded in encouraging handwashing behaviors in many children. Numerical visualization has been suggested to amplify children’s motivation.

## Data availability statement

The raw data supporting the conclusions of this article will be made available by the authors, without undue reservation.

## Ethics statement

The studies involving humans were approved by Ethical Board of Sapporo Medical University (1-2-22). The studies were conducted in accordance with the local legislation and institutional requirements. Written informed consent for participation was not required from the participants or the participants’ legal guardians/next of kin because it was implemented as part of school education.

## Author contributions

AO: Conceptualization, Data curation, Formal analysis, Funding acquisition, Investigation, Methodology, Project administration, Resources, Software, Supervision, Validation, Visualization, Writing – original draft, Writing – review & editing. YN: Conceptualization, Data curation, Investigation, Methodology, Supervision, Validation, Writing – review & editing. HY-S: Conceptualization, Supervision, Writing – review & editing. KN: Supervision, Writing – review & editing. MK: Supervision, Writing – review & editing. HO: Supervision, Writing – review & editing.
